# Lipid Metabolism is the common pathologic mechanism between Type 2 Diabetes Mellitus and Parkinson's disease

**DOI:** 10.7150/ijms.46456

**Published:** 2020-07-06

**Authors:** Xi Zhang, Yu Fan, Yuping Luo, Lingjing Jin, Siguang Li

**Affiliations:** 1 Stem Cell Translational Research Center, Tongji Hospital, Tongji University School of Medicine, Shanghai 200065, China.; 2 Key Laboratory of Spine and Spinal Cord Injury Repair and Regeneration of Ministry of Education, Orthopedic Department of Tongji Hospital, Tongji University School of Medicine, Shanghai 200065, China.; 3 Department of Neurology, Tongji Hospital, Tongji University School of Medicine, Shanghai 200065, China.

**Keywords:** bioinformatical analysis, Parkinson's disease, type 2 diabetes mellitus, biomarker, lipid metabolism

## Abstract

Although increasing evidence has suggested crosstalk between Parkinson's disease (PD) and type 2 diabetes mellitus (T2DM), the common mechanisms between the two diseases remain unclear. The aim of our study was to characterize the interconnection between T2DM and PD by exploring their shared biological pathways and convergent molecules. The intersections among the differentially expressed genes (DEGs) in the T2DM dataset GSE95849 and PD dataset GSE6613 from the Gene Expression Omnibus (GEO) database were identified as the communal DEGs between the two diseases. Then, an enrichment analysis, protein-protein interaction (PPI) network analysis, correlation analysis, and transcription factor-target regulatory network analysis were performed for the communal DEGs. As a result, 113 communal DEGs were found between PD and T2DM. They were enriched in lipid metabolism, including protein modifications that regulate metabolism, lipid synthesis and decomposition, and the biological effects of lipid products. All these pathways and their biological processes play important roles in both diseases. Fifteen hub genes identified from the PPI network could be core molecules. Their function annotations also focused on lipid metabolism. According to the correlation analysis and the regulatory network analysis based on the 15 hub genes, Sp1 transcription factor (SP1) could be a key molecule since it affected other hub genes that participate in the common mechanisms between PD and T2DM. In conclusion, our analyses reveal that changes in lipid metabolism could be a key intersection between PD and T2DM, and that SP1 could be a key molecule regulating these processes. Our findings provide novel points for the association between PD and T2DM.

## Introduction

Parkinson's disease (PD) is the second most common neurodegenerative disorder, causing irreversible, progressive motor and nonmotor dysfunction. PD is characterized by neuronal loss in the substantia nigra and other brain regions with the presence of intracytoplasmic protein inclusions known as Lewy bodies 1. Type 2 diabetes mellitus (T2DM) is a prevalent metabolic disorder characterized by obesity and chronic insulin resistance. T2DM comprises about 90% of all diabetic cases 2. However, the potential relationship between these two refractory diseases remains unclarified.

Recently, several clinical studies with conflicting results have given deeper insight into the association between T2DM and PD 3. Evidence from a case-control study suggested that diabetic individuals may have a decreased incidence of PD 4. However, data from a meta-analysis showed no conclusive evidence supporting T2DM as a risk factor for PD 5. In addition, some studies indicated that the contributing role of T2DM as a risk factor for PD could be associated with certain ethnic groups, including American 6, British 7, Danish 8, and Chinese 9. Meanwhile, it was reported that T2DM is most likely associated with PD progression 10,11, as evidenced by repeated inpatient care, longer duration of hospitalization 12, a higher United Parkinson's Disease Rating Scale (UPDRS), and more severe Hoehn and Yahr staging 13 in PD patients with T2DM. Notably, the conflicting results may be due to several confounding factors, one of which is the impact of drugs. While PD medications (such as levodopa) induce hyperglycemia and hyperinsulinemia 14, anti-diabetic drugs (such as metformin 15, glitazones 16, and exenatide 17) could elicit neuroprotection in PD and might lower the risk of acquired PD in populations with diabetes. In a word, the aforementioned findings highlight the detrimental impact that T2DM imposes on PD patients.

Even though T2DM is considered as a risk factor for developing PD, the exact mechanisms that explain the coexistence of these two disorders remains unclear. Recent studies have revealed convergent molecular and biological pathways that link both diseases. Hyperglycemia could be the most fundamental pathway. As previously reported, the majority of PD patients have abnormal glucose tolerance and display hyperglycemia 18, let alone T2DM. As a consequence of hyperglycemia, glycation is exacerbated in T2DM, and the formation of its agents is increased 19. Accordingly, increased levels of glycation have been reported in the cerebral cortex, amygdala, and substantia nigra of PD patients 20. Besides, chronic hyperglycemia is often accompanied by insulin signaling disorders that are common in both PD and T2DM patients. In turn, insulin signaling disorders can also alter systemic glucose and lipid metabolism, and impaired lipid metabolism is associated with inflammatory, oxidative stress, mitochondrial dysfunction, and insulin resistance 3. Moreover, amyloid formation is another shared mechanism. One of the typical pathological features in PD is proteinaceous amyloid fibrils, which are composed mostly of alpha-synuclein, called Lewy pathology 1. In T2DM, another disease involving amyloid formation, the primary pathological characteristic is islet amyloid polypeptide in pancreatic β-cells 21. The amyloid formation initiates many processes, including endoplasmic reticulum stress, unfolding protein response, oxidative stress, autophagy, mitochondrial dysfunction, and cell apoptosis 3. In general, the various processes do not exist independently but rather influence each other. They are reciprocal causations in the two disorders.

Common transcriptional signatures may provide further insight into the shared biological mechanisms in PD and T2DM. In the current study, we compared the expression profiles in T2DM and PD, and analyzed the communal differentially expressed genes (DEGs) in order to identify common pathologic mechanisms and protein‐protein interaction (PPI) nodes.

## Materials and Methods

### Data preprocessing and identification of DEGs

**Figure 1:**
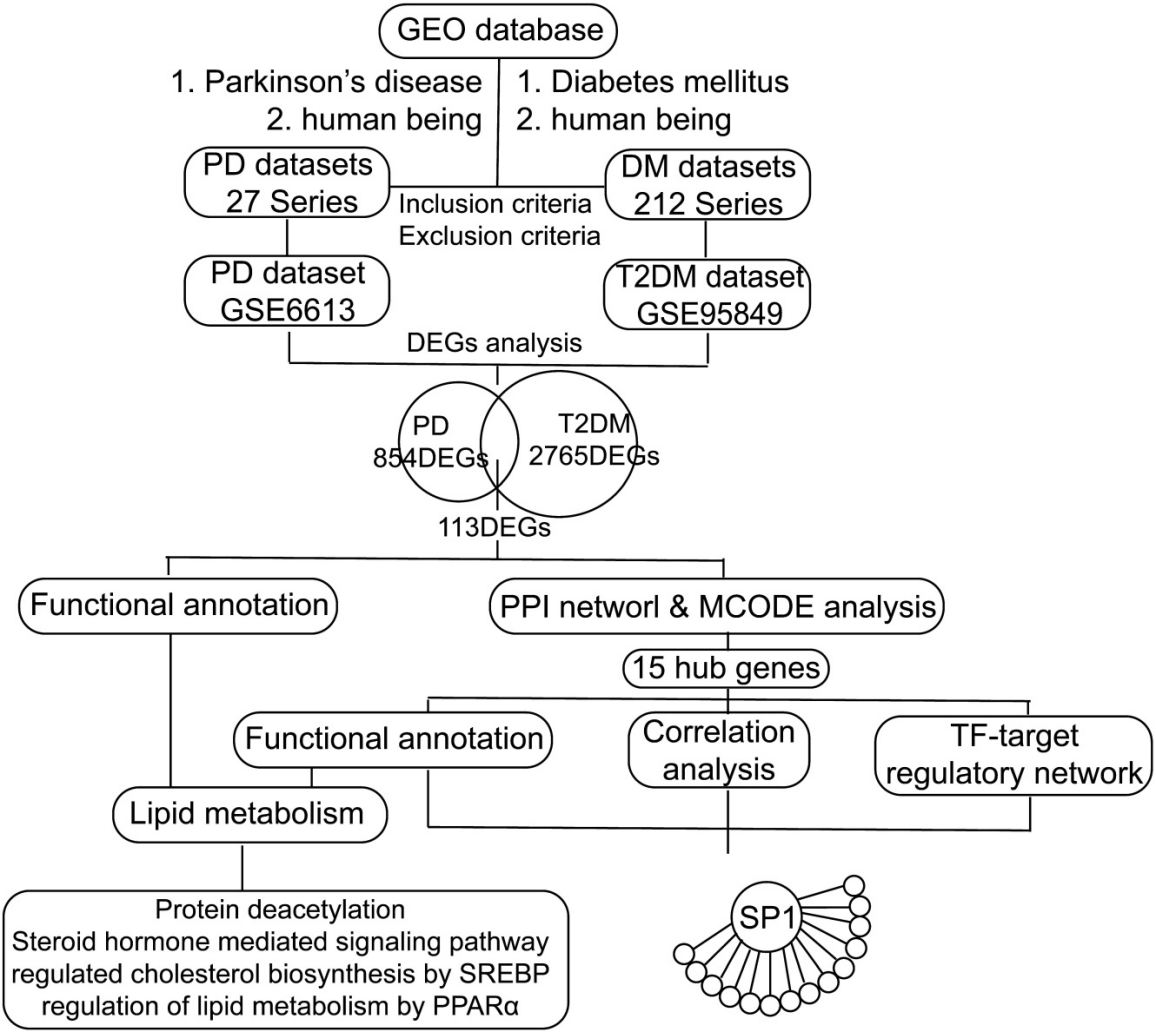
Flow chart of the study. GEO: Gene Expression Omnibus, PD: Parkinson's disease, DM: diabetes mellitus, T2DM: type 2 diabetes mellitus, DEGs: differentially expressed genes, PPI: protein-protein interaction, TF: transcription factor, SREBP: sterol regulatory element binding protein, PPARα: peroxisome proliferator activated receptor alpha, SP1: Sp1 transcription factor.

We retrieved transcription profile datasets of PD and T2DM from the NCBI Gene Expression Omnibus (GEO) database 20 based on the keywords "Parkinson's disease and human being" and "Diabetes mellitus and human being". These datasets were screened based on inclusion/exclusion criteria. The inclusion criteria were as follows: 1. Sporadic PD or type 2 diabetes mellitus; 2. Transcriptional RNA expression profiles based on peripheral blood; 3. Datasets that included patients and healthy controls. The exclusion criteria were as follows: Patients had participated in a clinical trial for drugs or other treatments. Finally, the transcription profiles in peripheral blood in PD (GSE6613, including 50 PD patients and 23 controls) and T2DM (GSE95849, including 6 T2DM patients and 6 controls) were obtained (Figure 1). According to the platforms, preprocessing and normalization of raw data (CEL files) were carried out with the affy package (version 1.64.0) 22 and the limma package (version 3.42.2) 23. After excluding patients with other diseases, the following processes were performed: data format conversion, missing value imputation, background adjustment, quantile normalization, and principal components analysis (PCA) 24. According to the PCA results, we excluded one sample affecting classification and reserved five T2DM patients and six healthy controls (HCs) in GSE95849. Since all the samples of GSE6613 were jumbled together and could not be classified at all according to the PCA and the clustering results, we randomly selected PD patients and HCs to avoid errors caused by subjective factors. And we decided to randomly selected six PD patients and six HCs, the same as the sample size of GSE95849. Significant expression differences were analyzed using the Bayesian analysis method provided by the limma package 23 with the cut-off value p < 0.05 and │Log_2_fold change (FC)│ > 1.0. In the following study, the intersection of the 2-dataset DEGs was defined as communal DEGs and visualized by the VennDiagram package (version 1.6.20) 25.

### Functional annotation of significant DEGs

Gene ontology (GO), Kyoto Encyclopedia of Genes and Genomes (KEGG) pathways and Reactome pathways enriched by DEGs were analyzed by the clusterProfiler package (version 3.14.0) 26 and the ReactomePA package (version 1.30.0) 27 with the significant selection criteria including a p value <0.05 and gene count ≥ 2.0.

### Integration of the PPI network and correlation analysis of hub nodes

The Search Tool for the Retrieval of Interacting Genes (STRING) online tool 28 was applied to analyze the PPI of the communal DEGs with the threshold of the combined score >0.15. The PPI network was constructed using the Cytoscape software (version 3.7.1) 29. To identify hub nodes with higher betweenness centrality (BC), we used CytoHubba plug-in 30 in Cytoscape to analyze the significant nodes in the PPI network. In addition, the most highly interconnected module in the PPI network was analyzed using the plug-in MCODE 31 with default parameters. Correlations of hub genes with higher BC and hub nodes in MCODEs were calculated and visualized by the ggplot2 package (version 3.3.1).

### Transcription factor-target regulatory network

The transcription factors (TFs) that target hub genes in the PPI network were predicted using the Cytoscape plug-in iRegulon 32, which integrates information from the lager motif and track collections. We obtained the data predicted by the track discovery of existing regulatory datasets, which include data validated by ChIP-Seq, DHS-seq, or FAIRE-seq. TF-target pairs with normalized enrichment scores (NES) >4 were selected.

## Results

### Identification of 113 communal DEGs between PD and T2DM

**Figure 2:**
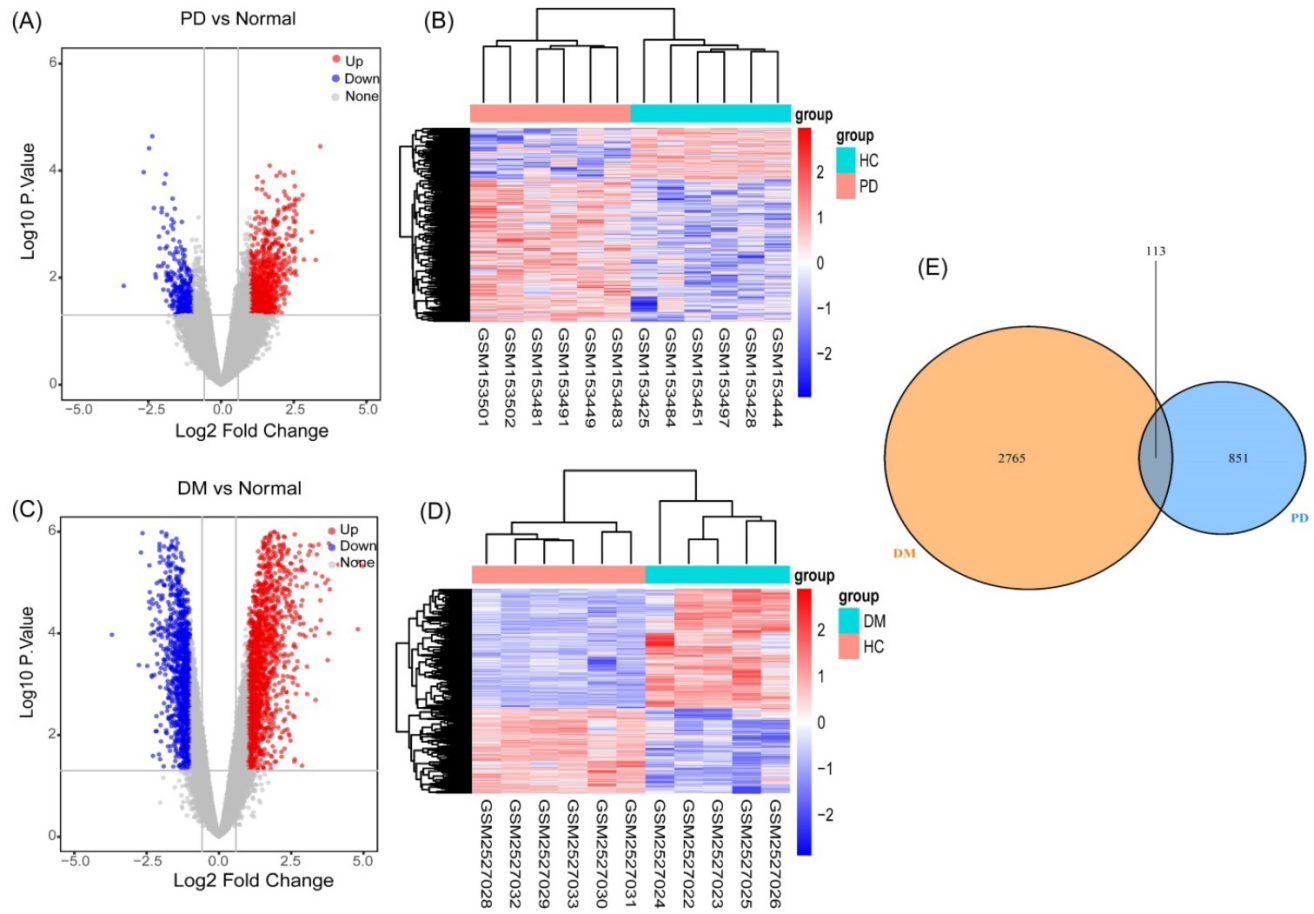
Identification of gene expression profiles in the two datasets. (**A**) Volcano plot of PD microarray data. (**B**) The cluster heat map of PD DEGs. (**C**) Volcano plot of T2DM microarray data. (**D**) The cluster heat map of T2DM DEGs. (**E**) Venn diagram of the 194 communal DEGs between T2DM and PD.

As described in the materials and methods section, based on the PCA and the clustering analyses, samples affecting classification were excluded from the preprocessed datasets (Figure S1, S2). We performed differential gene analysis and cluster analysis based on the screened samples, which included six PD patients and six HCs in GSE6613, and five T2DM patients and six HCs in GSE95849. We identified 964 genes (694 upregulated and 270 downregulated genes) (Figure 2A) as significant DEGs in patients with PD, compared to the controls. Meanwhile, compared to the controls, 2878 genes (1686 upregulated and 1192 downregulated genes) (Figure 2C) were identified as DEGs in T2DM. The results of the cluster analysis (Figure 2B, 2D) showed significant differences in the DEGs in PD and T2DM. By analyzing the shared DEGs of PD and T2DM, we found 113 communal DEGs (Figure 2E), including 49 co-upregulated and 12 co-downregulated genes.

### Communal DEGs between PD and T2DM focused on the regulation of lipid metabolism

**Figure 3:**
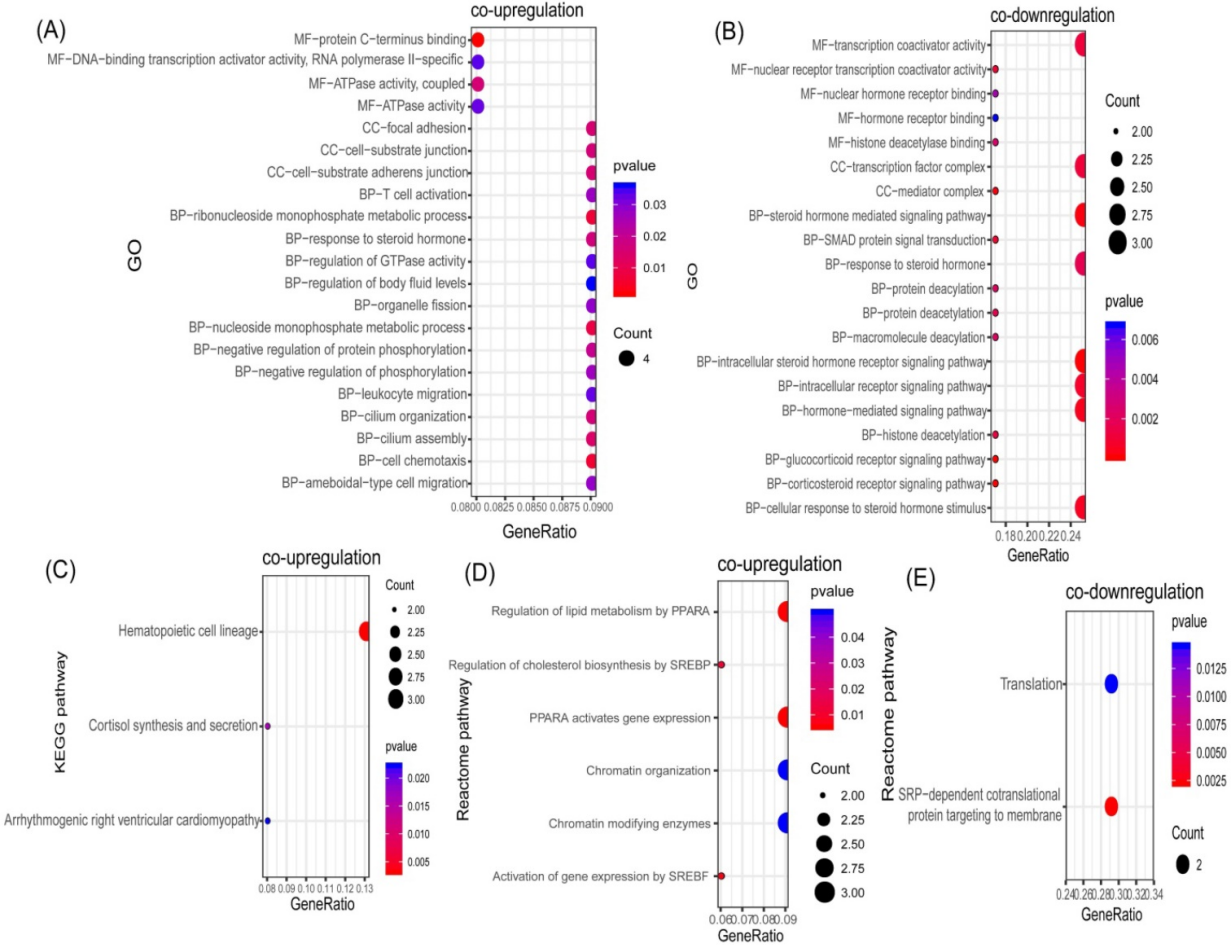
Functional annotation of communal DEGs. (**A**) Bubble plot of the Gene ontology (GO) enriched by co-upregulated DEGs. (**B**) Bubble plot of the GOs enriched by co-downregulated DEGs. (**C**) Bubble plot of the KEGG pathway enriched by co-upregulated DEGs. (**D**) Bubble plot of the Reactome pathway enriched by co-upregulated DEGs. (**E**) Bubble plot of the Reactome pathway enriched by co-downregulated DEGs

In order to identify potential communal biological processes and pathways between PD and T2DM, functional annotation analyses were performed based on the communal DEGs identified. According to our selection criteria, three KEGG pathways, six Reactome pathways, and 21 GOs were enriched by co-upregulated genes (Figure 3A, 3C, 3D), while two Reactome pathways and 20 GOs were enriched by co-downregulated genes (Figure 3B, 3E). The co-upregulated DEGs were enriched mainly in processes and pathways associated with the regulation of cholesterol biosynthesis (through the sterol regulatory element binding protein (SREBP)) and the regulation of lipid metabolism (through peroxisome proliferator activated receptor alpha (PPARα)). The co-downregulated DEGs were enriched mainly in deacetylation modifications and the steroid hormone mediated signaling pathway. These results suggested that the regulation of lipid metabolism could be the common pathologic mechanism that links both diseases.

### Hub nodes in the PPI network focused on lipid metabolism processes

**Figure 4:**
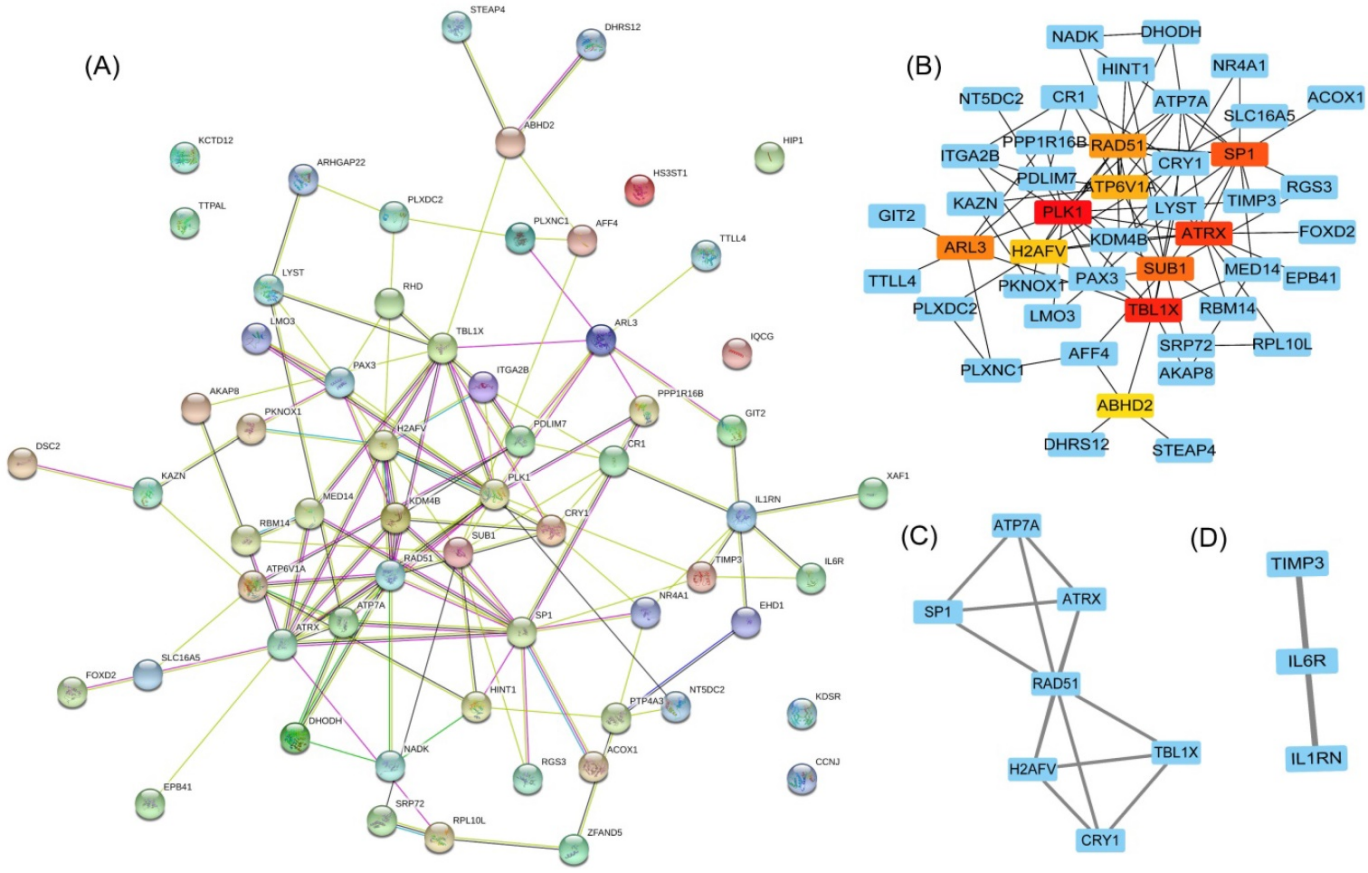
Hub gene identification in a PPI network based on communal DEGs. (**A**) The PPI network of co-upregulated and co-downregulated DEGs. (**B**) The top 10 node genes in the PPI network. (**C**) Cluster 1 analyzed by the plug-in MCODE in the whole PPI network. (**D**) Cluster 2 analyzed by the plug-in MCODE in the whole PPI network.

**Figure 5:**
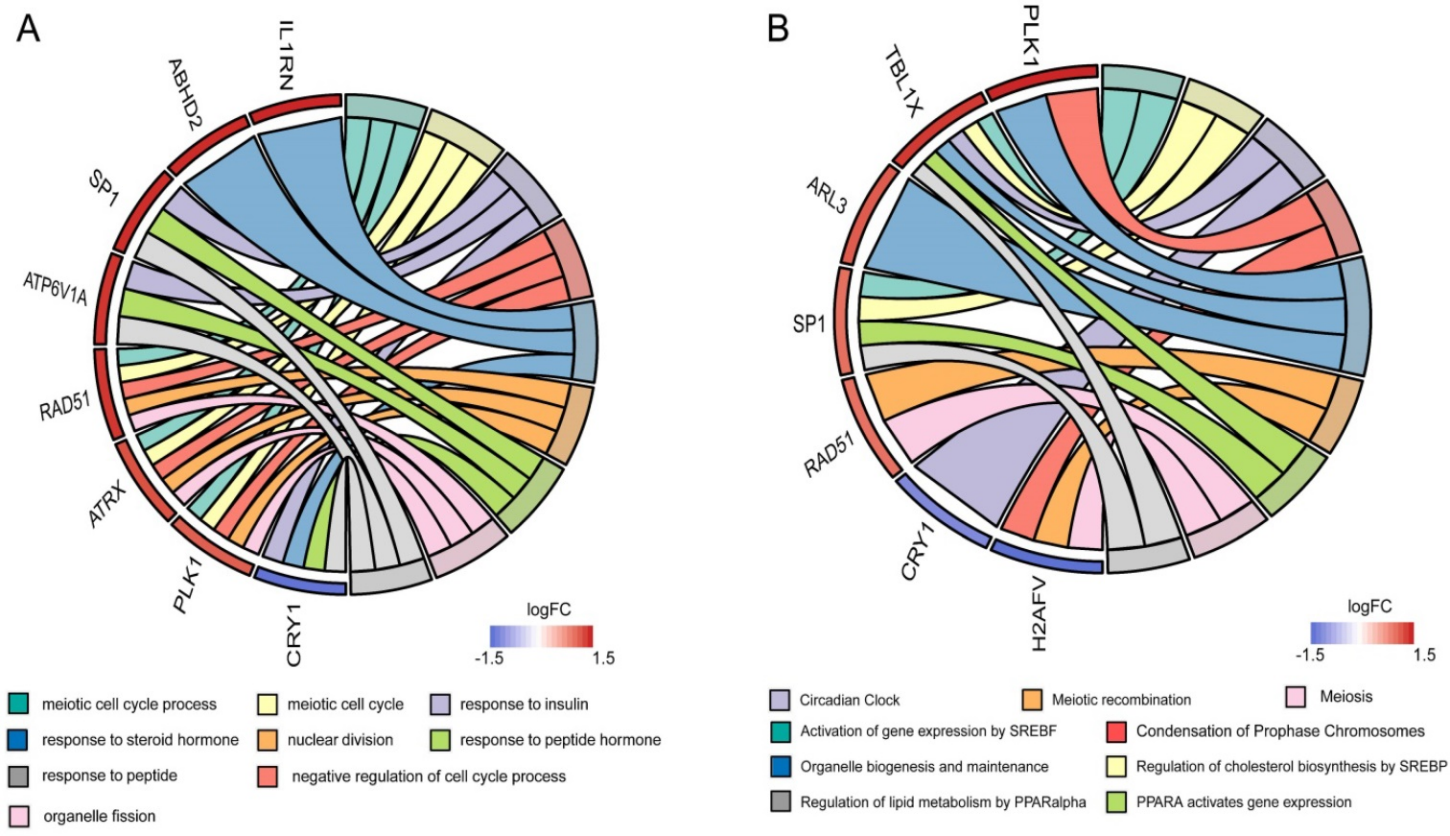
Functional annotation of the hub genes. (**A**) Circos plot of the GOs enriched by hub genes. (**B**) Circos plot of the Reactome pathway enriched by hub genes.

To clarify the interactions between DEGs, we performed a PPI network analysis on all communal DEGs (Figure 4A), and predicted core genes in the network through the topological property of BC (Figure 4B, Supplementary Table 1) and clustering algorithms called MCODE (Figure 4C, 4D). Combining the results of these two analyses, 15 key genes were obtained, in which Sp1 transcription factor (SP1), RAD51 paralog B (RAD51), transducin (beta)-like 1 X-linked (TBL1X), H2A histone family, member V (H2AFV), and alpha-thalassemia/mental retardation syndrome X-linked (ATRX) were identified by both methods. To verify the identified biological processes shared between PD and T2DM, function annotations of the 15 hub genes were performed. The results of those studies highlighted metabolic processes such as the response to insulin and steroid hormones, the activation of gene expression by SREBF, and the activation of gene expression by PPARα (Figure 5). The function annotations of the 15 hub genes were similar to the results of all 113 common DEGs, enhancing the credibility of the conclusion that lipid metabolism processes are the common pathologic mechanism between PD and T2DM.

### SP1 was identified as a key molecule that affects other hub genes

**Figure 6:**
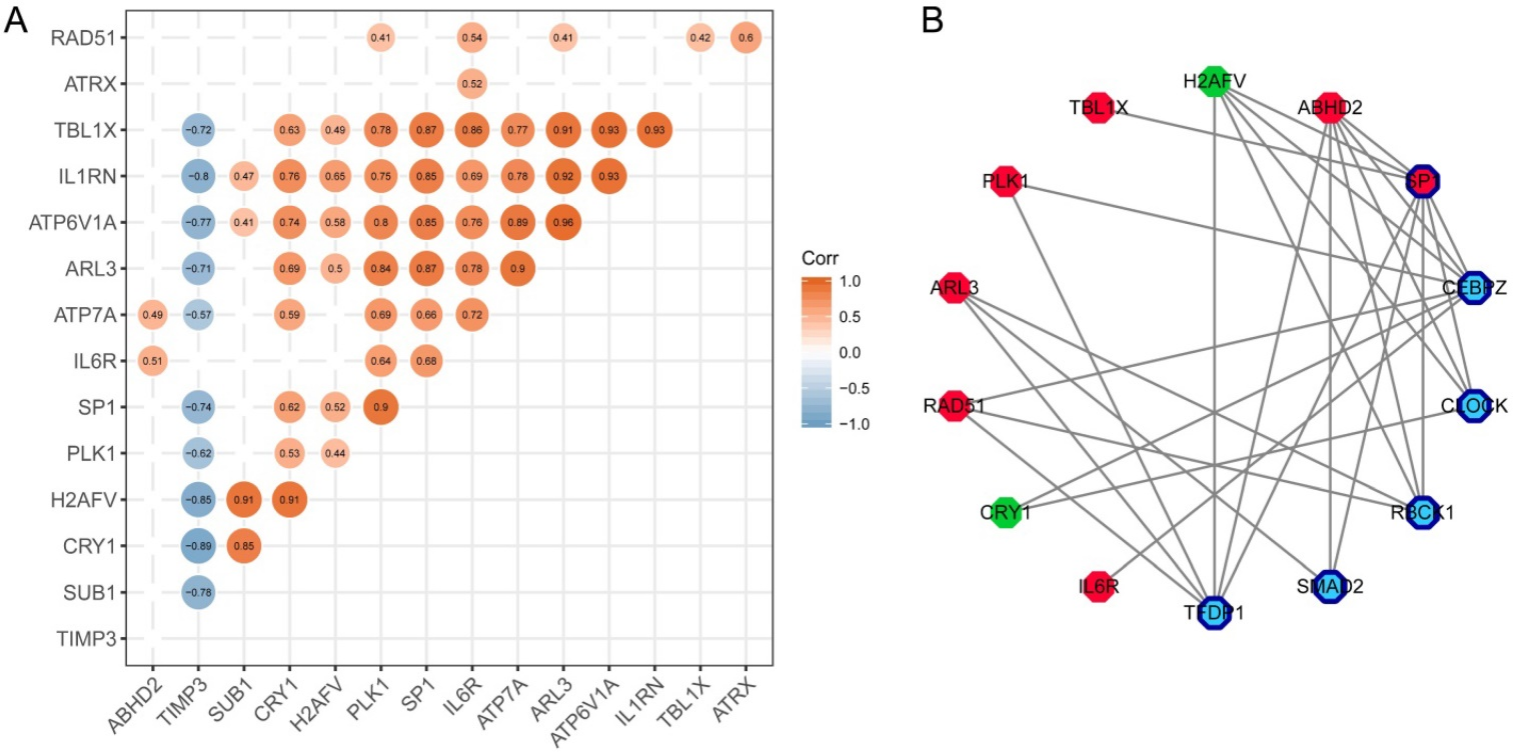
Identification of the key molecule affecting other hub genes. (**A**) The correlation analysis between hub genes. (**B**) TF-targeting regulatory network based on hub genes. Red polygons represent upregulated hub genes; green polygons represent downregulated hub genes; dark blue bordered polygons represent transcription factors; blue polygons represent predicted transcription factors.

To find the most important regulatory molecule in the 15 hub genes, we analyzed correlations between the hub genes (Figure 6A) and constructed the TF-target regulatory network (Figure 6B, Supplementary Table 2). While the majority of the hub genes had significant correlations with each other, SP1 had either direct or indirect interactions with the other genes in the TF-target regulatory network. In other words, SP1 could be a key gene that regulates and connects with the other 14 hub genes. It is a nuclear transcription factor that can activate or repress the transcription of many genes. We reviewed the roles of the genes in the TF-target network in T2DM and PD (Table 1).

## Discussion

The last decade has witnessed an unceasing debate on the potential relationship between PD and T2DM. We attempted to associate the transcriptome data of T2DM patients with PD patients in order to explore the crosstalk between the two diseases.

In our study, all of the functional annotation results can be summed up in three aspects: protein modifications (protein deacetylation), lipid synthesis and decomposition (the regulation of cholesterol biosynthesis by SREBP and the regulation of lipid metabolism by PPARα), and the biological effects of lipid products (steroid hormone-mediated signaling pathways). Firstly, protein acetylation and deacetylation are posttranslational protein modifications that regulate inflammation, oxidative stress, mitochondrial function, and glucose and lipid metabolism in PD 33 and T2DM 34. Secondly, overactivated SREBP means an imbalance between cholesterol synthesis and decomposition, while changes in the activity of PPARα can break the balance in lipid oxidation. All of these disorder processes not only contribute to α-synuclein aggregation and dyslipidemia 35,36, but also lead to elevated levels of both lipid and protein oxidation in T2DM and PD 37,38. Finally, steroid hormones may participate in both PD 39 and T2DM 40. For example, endogenous sexual hormones might have neuroprotective effects against neurotoxic agents for dopamine neurons 41, and modulate the glycemic status and risk of T2DM 42.

According to our analyses, 15 hub nodes were identified in the PPI network. The functional annotation results of these hub nodes also highlighted lipid metabolism, and showed these nodes to have significant correlations with each other. In the TF-target regulatory network, SP1 was the core factor that regulated the other genes either directly or through predicted transcription factors. SP1 not only regulates insulin signaling and cholesterol metabolism 43, but it is also a common interpreter of nuclear signal transduction in response to hormones 44. Besides, SP1 modulates the expression and activity of PD-related genes to produce neuroprotective effects 45,46. In conclusion, SP1 and its relevant genes may act as a core intersection in the crosstalk between T2DM and PD.

Santiago and Potashkin 47 discussed the pathways that are shared between PD and T2DM and obtained a key shared gene, amyloid precursor protein. The results were based on the analyses of confirmed genes associated with PD and T2DM. These genes were obtained from the genome-wide association studies (GWAS) catalog that collected published GWAS analyses, and were then verified by GEO microarray studies and clinical samples. GWAS aims at identifying genetic variants and disease-trait associations, but cannot identify all genetic determinants of complex traits 48. Besides, complex traits and complex diseases, such as PD and T2DM, can be attributed not only to genotype but also environment. Results based solely on GWAS data do not include the substantial contribution of environmental factors. It is preferable to combine GWAS data with microarray studies and validation using clinical samples. In our study, we chose to analyze gene expression data from sporadic PD and T2DM patients in order to analyze the transcriptional response of the genome to environmental stimuli or physiological/pathological conditions 49.

Several limitations in our study should be acknowledged. First, the hub nodes identified need to be validated in future studies. Second, the sample size in this study was relatively small, and external validation is needed to consolidate our results. Additionally, it is necessary to perform functional studies to confirm the roles of the DEGs in T2DM and PD. Future functional verification could be performed on model organism that could be used to explore pathway-/gene-disease associations by gain or loss of function.

In conclusion, the communal DEGs and pathways identified in our study reveal transcriptome links between T2DM and PD, and provide novel mechanisms and targets that involve changes in lipid metabolism. Additionally, SP1 could be a key molecule regulating these processes.

## Supplementary Material

Supplementary figures and tables.

## Figures and Tables

**Table 1:** Review of the hub nodes in the transcription factor (TF)-target regulatory network

Genes	T2DM	PD
polo-like kinase 1 (PLK1)	Stimulation of KLF14/PLK1 pathway potentiates endothelial dysfunction in Type 2 diabetes mellitus 50.	PLK1 is involved in the phosphorylation of aggregated α-syn at S129 in this system; knockdown of PLK1 significantly inhibit Cory-induced autophagy that promotes the clearance of PD-associated SNCA/α-synuclein 51.
transducin (beta)-like 1X-linked (TBL1X)	None	None
Sp1 transcription factor (SP1)	The specific recognition of -420G by Sp1/3 increases RETN promoter activity, leading to enhanced serum resistin levels, thereby inducing human T2DM 52.	SP1 is a principal factor regulating increases in MAO B activity, and SP1 inhibition produces neuroprotective effects in PD models through decreases in MAO B activity, which may be a new neuro-protective therapeutic strategy for PD treatment 46.
ADP-ribosylation factor-like 3 (ARL3)	None	None
RAD51 paralog B (RAD51)	Advanced Glycation End‐Products decrease the expression of RAD51 and RAD52 in INS-1 cells 53.	DNA repair proteins like p-CREB, APE1 and Rad51 were increased in response to rotenone-induced DNA damage 54.
H2A histone family, member V (H2AFV)	None	None
abhydrolase domain containing 2 (ABHD2)	None	None
Circadian Regulator 1 (CRY1)	Insulin-activated SREBP1c downregulates gluconeogenesis through CRY1-mediated FOXO1 degradation and dysregulation of hepatic SREBP1c-CRY1 signaling may contribute to hyperglycaemia in diabetic animals 55.	None
Interleukin 6 Receptor (IL6R)	IL6R inhibits viability and apoptosis of pancreatic beta-cells in type 2 diabetes mellitus via regulation by miR-22 of the JAK/STAT signaling pathway 56.	Protein expression of IL-1R, IL-6R, and TNFR subtype TNFR1 in the plasma membrane midbrain periaqueductal gray of PD rats was upregulated 57.
