# Supplementary material for: Lipid Metabolism is the common pathologic mechanism between Type 2 Diabetes Mellitus and Parkinson's disease
Source: Int J Med Sci. 2020 Jul 6;17(12):1723–32. doi: 10.7150/ijms.46456 (PMC7378658; doi:10.7150/ijms.46456)
Supplement: Supplementary file 1 — Supplementary figures and tables. [file ijmsv17p1723s1.pdf]

Supplementary Figures

Supplementary Figure 1

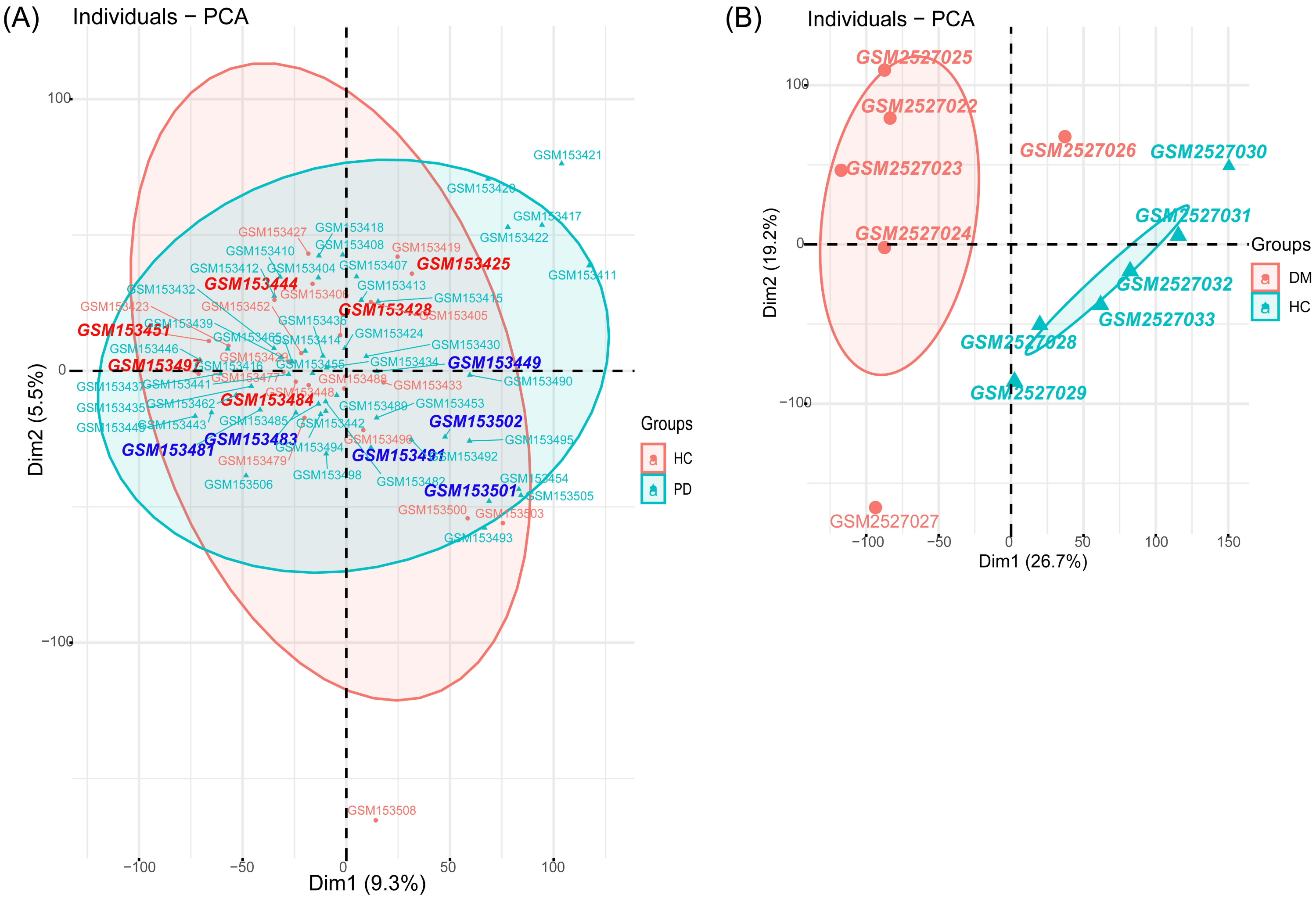

Supplementary Figure 2

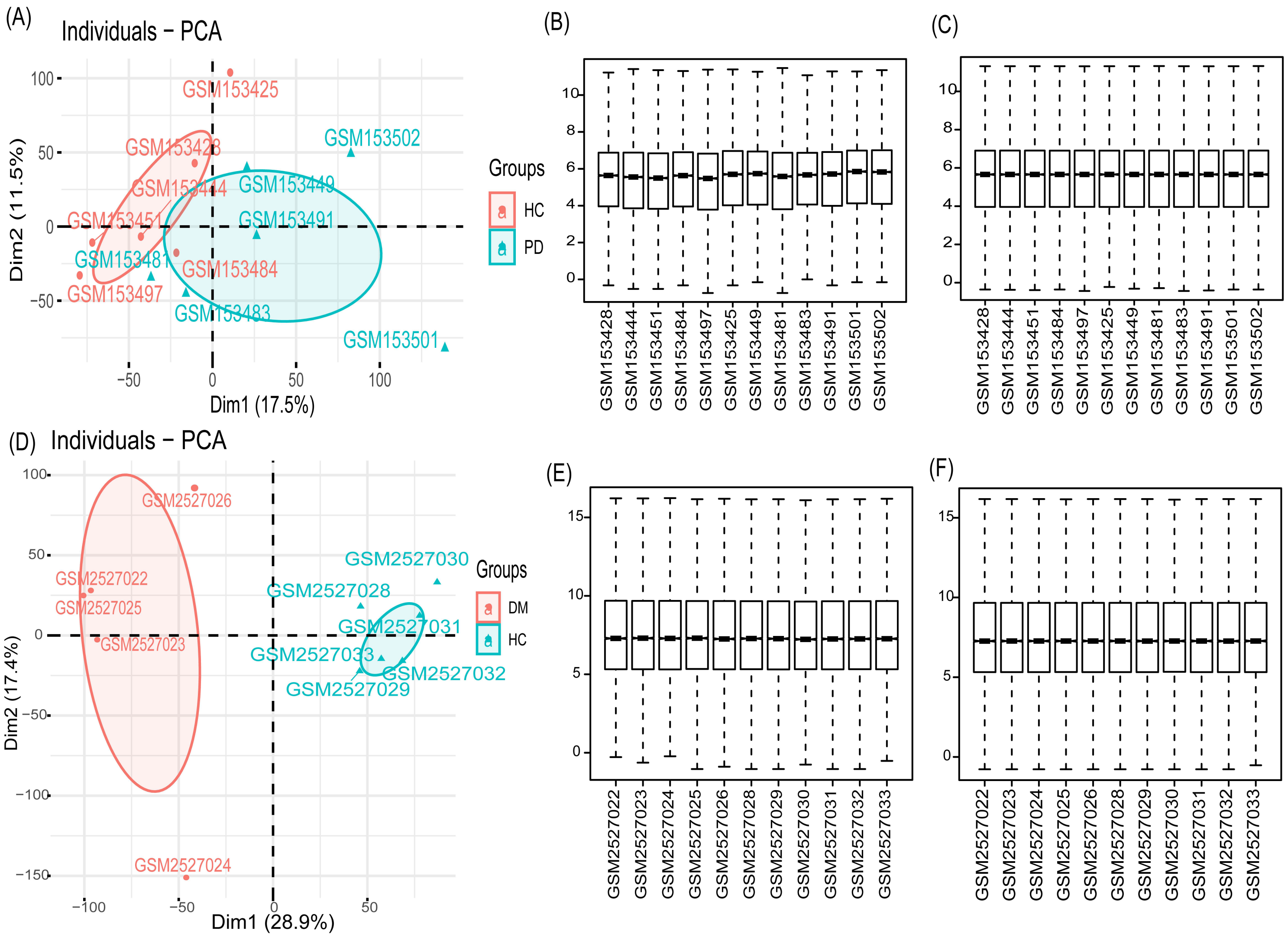

Supplementary Tables

Supplementary Table 1. The score of 15 hub nodes in the PPI network

**Genes**	**Betweenness**	**Closeness**	**Degree**
PLK1	541.7583	13	31.08333
TBL1X	409.64957	9	27.91667
ATRX	386.27446	11	28.58333
SP1	364.5728	12	28.91667
SUB1	347.06479	9	28.25
ARL3	305.37381	7	25.75
RAD51	265.42027	11	29.16667
ATP6V1A	257.97857	6	24.16667
H2AFV	238.23874	9	28
ABHD2	213.33333	4	20.68333
TIMP3	143.51299	4	22.95
ATP7A	136.6597	7	26.33333
IL1RN	132.8913	5	20.38333
CRY1	91.03911	6	24.91667
IL6R	0	2	17.33333

Supplementary Table 2. The predicted TFs of hub nodes in the PPI network

Track id	NES	Transcription factor	Target genes
wgEncodeHaibTfbsHct116Sp1V0422111PkRep1.broadPeak.gz	5.28449	SP1	SP1,H2AFV,ABHD2,TBL1X
GSM1208719_batch2_chrom1_LoVo_CLOCK_PassedQC_peaks_hg19	5.07536	CLOCK	SP1,ABHD2,CRY1,H2AFV
GSM1208691_batch1_chrom1_LoVo_TFDP1_PassedQC_peaks_hg19	4.88838	TFDP1	H2AFV,AD51,ARL3,PLK1,SP1,ABHD2
wgEncodeSydhTfbsHepg2CebpzIggrabPk.narrowPeak.gz	4.7137	CEBPZ	SP1,ABHD2,H2AFV,IL6R,PLK1,CRY1,RAD51
GSM1208789_batch2_chrom1_LoVo_RBCK1_PassedQC_peaks_hg19	4.33481	RBCK1	SP1,ABHD2,H2AFV,ARL3,RAD51
GSM1208674_batch1_chrom1_LoVo_SMAD2_PassedQC_peaks_hg19	4.11338	SMAD2	SP1, ABHD2, ARL3
